# RANK receptor oligomerisation in the regulation of NFκB signalling

**DOI:** 10.1530/JME-14-0077

**Published:** 2014-08

**Authors:** S Das, I Sepahi, A Duthie, S Clark, J C Crockett

**Affiliations:** 1 Bone and Musculoskeletal Research Programme, Division of Applied Medicine Institute of Medical Sciences, University of Aberdeen Foresterhill, Aberdeen, AB25 2ZD UK

**Keywords:** oligomerisation, RANK, NFκB, osteopetrosis

## Abstract

The interaction of receptor activator of NFκB (RANK), a member of the tumour necrosis factor receptor superfamily, with RANK ligand is crucial for the formation, function and survival of osteoclasts. The role of the cytoplasmic oligomerisation domain (pre-ligand assembly domain; PLAD or ‘IVVY’ motif) in the ligand-dependent activation of downstream NFκB signalling has not been studied previously. The discovery of truncating mutations of *TNFRSF11A* (W434X and G280X that lack the PLAD) as the cause of rare cases of osteoclast-poor osteopetrosis offered the opportunity for functional study of this region. Recapitulating the W434X mutation by transcription activator-like effector nuclease (TALEN)-mediated targeted disruption of *Tnfrsf11a* within the region homologous to W434X in the mouse macrophage-like cell line RAW264.7 impaired formation of osteoclast-like cells. Using overexpression studies, we demonstrated that, in contrast to WT-RANK, the absence of the PLAD in G280X-RANK and W434X-RANK prevented ligand-independent but not ligand-dependent oligomerisation. Cells expressing W434X-RANK, in which only two of the three TRAF6-binding motifs are present, continued to exhibit ligand-dependent NFκB signalling. Hence, the absence of the PLAD did not prevent ligand-induced trimerisation and subsequent NFκB activation of RANK, demonstrating that therapeutic targeting of the PLAD in the prevention of osteoporosis may not be as effective as proposed previously.

## Introduction

The interaction of receptor activator of NFκB (RANK; encoded by *TNFRSF11A*) with RANK ligand (RANKL; encoded by *TNFSF11*) is critical for the formation and function of osteoclasts (Anderson *et al*. 1997), and the absence of either RANK or RANKL in mice results in a severe osteopetrotic phenotype (Dougall *et al*. 1999, Kong *et al*. 1999). Recent papers have described a total of 12 mutations within *TNFRSF11A* that cause a severe form of recessive osteopetrosis (Guerrini *et al*. 2008, Pangrazio *et al*. 2012). In the majority of cases, the patients present in early infancy with a range of complications with or without bone fractures. Bone marrow biopsies show paucity of multinucleated osteoclasts.

RANK is a member of the tumour necrosis factor receptor (TNFR) superfamily and shares many structural similarities with other members of this family. The functional characterisation of TNFR revealed that ligand-induced receptor oligomerisation is the first step towards the activation of downstream signal transduction (Tartaglia & Goeddel 1992). Subsequently, it was established that TNFR could also self-assemble into trimers when overexpressed in the absence of ligand (Boldin *et al*. 1995). These findings led to a search for specific domains in TNFR responsible for ligand-independent self-assembly. Chan *et al*. (2000) discovered the existence of such a domain in the extracellular region of the TNFR and introduced the term pre-ligand assembly domain (PLAD). Given that RANK is a TNFR family member, it was expected to contain a PLAD. Indeed, Kanazawa and Kudo identified a region within the cytoplasmic domain that seemed to behave as a PLAD (^534^I-I-V-V-Y-V^539^ in mice; ^545^I-I-V-V-Y-V^550^ in humans) (Kanazawa & Kudo 2005). Deletion of the PLAD prevented ligand-independent oligomerisation, and it was identified as a potential therapeutic target in osteoporosis. This sequence motif was shown to be important for the mediation of osteoclast formation *in vitro* (Xu *et al*. 2006).

All RANK mutations causing osteopetrosis identified to date are single-base substitutions or insertions. Two of these (c.838G>T (p.G280X) and c.1301G>A (p.W434X)) result in the introduction of premature stop codons. Mononuclear cells from a patient with the G280X mutation did not form multinucleated osteoclasts when cultured in the presence of macrophage colony-stimulating factor (M-CSF) and RANKL, while the bone marrow biopsy from a patient carrying the W434X mutation showed the absence of osteoclasts (Guerrini *et al*. 2008), indicating that both mutations have a catastrophic effect on the formation of osteoclasts.

**Figure 1:**
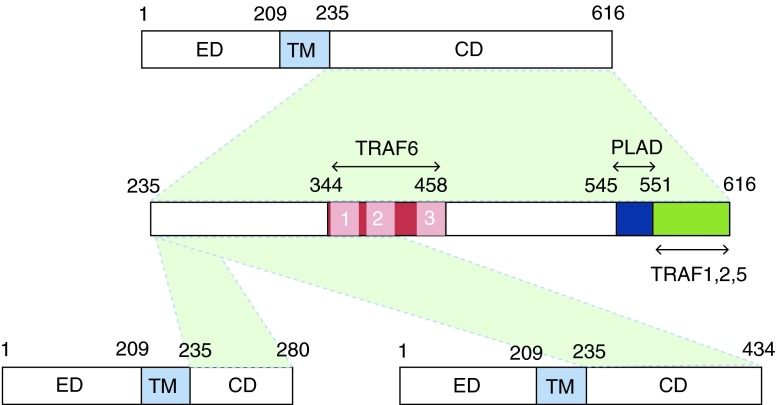
Schematic diagram of the intracellular domain of RANK. Full-length RANK, a single-pass receptor protein of 616 amino acids, with an extracellular domain (ED), transmembrane domain (TM) and cytoplasmic domain (CD). The numbers indicate amino acid sequence positions of each domain. The cytoplasmic domain shows the TRAF6-binding sites, the PLAD and other TRAF-binding sites located at the distal end of the receptor. The regions of the cytoplasmic domain present in each of the G280X and W434X mutant RANK proteins are shown.

Although both mutations lead to severe osteopetrosis, the way in which they affect osteoclast differentiation may differ. Both mutations result in the loss of a significant proportion of the distal end of the RANK receptor. In W434X-RANK, the PLAD motif and one recognised TRAF6-binding motif (p.453–458) is absent, whereas in G280X-RANK both the PLAD and all the three conserved TRAF6-binding motifs (equivalent to human amino acid positions p.344–349, p.377–382 and p.453–458 (Darnay *et al*. 1998)) are absent (Fig. 1). The aim of this study was to dissect the functional consequences of these truncating mutations to provide information on the molecular basis for the patient phenotypes and provide new insights into the domains regulating RANK signalling in humans.

## Materials and methods

### Cell lines and reagents

HEK293, HeLa and RAW264.7 cell lines were obtained from the European Cell and Culture Collection (ECACC, Salisbury, UK). HEK293 cells were maintained at 37 °C in 5% CO_2_ in alpha-modified minimal essential medium (α-MEM) supplemented with 10% [v/v] FCS and 1 mM glutamine. HeLa and RAW264.7 cells were maintained at 37 °C in 5% CO_2_ in DMEM supplemented with 10% [v/v] FCS and 1 mM glutamine. All other reagents were purchased from Sigma-Aldrich, unless stated otherwise.

### Transcription activator-like effector nucleases design

**Figure 2:**
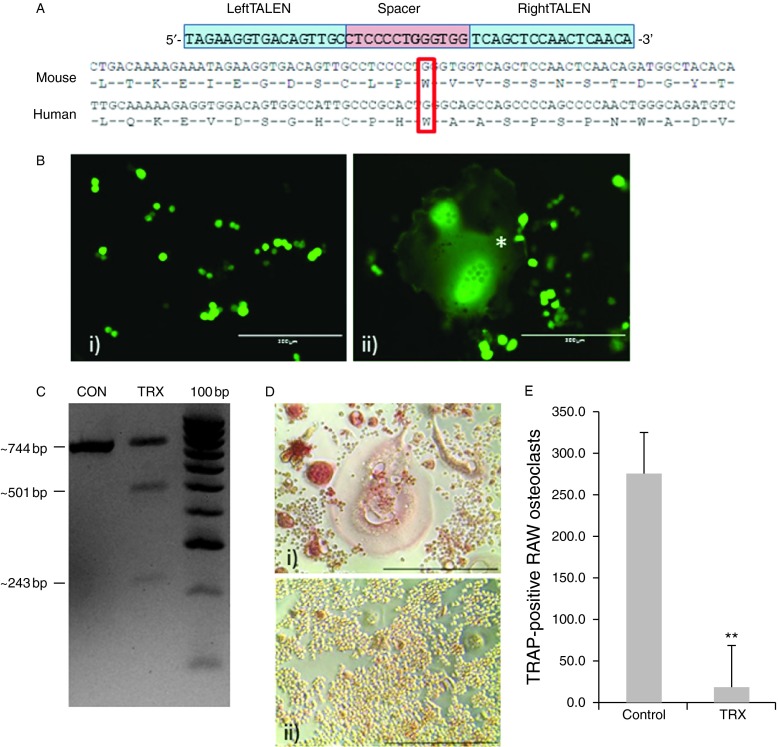
TALEN-based disruption of the *Tnfrsf11a* gene in RAW264.7 cells. (A) TALEN design, showing the target of the left and right TALENs centred at the G highlighted in bold corresponding to c.G1301A in human RANK, with alignment with mouse sequence performed in ClustalW (Chenna *et al*. 2003). (B) GFP expression 72 h after transfection in RAW264.7 cells in the i) absence and ii) presence of RANKL. (C) Detection of TALEN-mediated cut and base mismatch by Surveyor nuclease: expected product sizes of 744, 501 and 243 bp. (D) TRAP staining of i) untransfected and ii) TALEN-transfected RAW264.7 cells. Scale bars=400 μm. (E) Quantification of the number of TRAP-positive osteoclast-like cells in control and TALEN-transfected cultures. Data are means±s.d. of three independent experiments; **P<0.001, significantly different from control (Student's *t*-test).

Transcription activator-like effector nucleases (TALEN) constructs were custom-designed by Dundee Cell Products Ltd, Dundee, UK, against mouse RANK (*Tnfrsf11a* ENSMUSG00000026321) to introduce an exon 9 cut site (spacer sequence CTCCCCTGGGTGG) equivalent to the human RANK-W434X mutation site (Fig. 2A). The left TALEN (TAGAAGGTGACAGTTGC)- and right TALEN (TCAGCTCCAACTCAACA)-targeting sequences were constructed in SP6 vectors, and the expression of the TALENs was driven by a CMV promoter. The left and right TALEN sequences were sequence verified.

### Transfection of RAW264.7 cells

RAW264.7 cells were seeded into 10 cm Petri dishes at a density of 1×10^6^ cells. The cells were removed by trypsin/EDTA digestion on the following day. The cells were washed once with calcium and magnesium-free PBS and resuspended in 100 μl of Buffer R (Neon microporator, Life Technologies). The resuspended cells were mixed with 2.5 μg each of the left and right TALEN plasmids (Dundee Cell Products) or 2.5 μg of pMaxGFP (Lonza BioSciences, Cologne, Germany), and electroporation was performed on a Neon microporator according to the manufacturer's protocol for the transfection of RAW264.7 cells (pulse voltage 1680 V, pulse width 20 ms, and pulse number 1). The transfected cells were seeded in six-well plates or 48-well plates at a density of 3×10^5^ cells or 2×10^4^ cells/well respectively.

### Surveyor nuclease assay

The transfected cells were seeded in six-well plates and cultured for 3 days. Exon 9 of the RANK gene was amplified from purified genomic DNA using within-exon primers (forward primer: 5′-AGTCCTCAGGGGACCGTTGT-3′ and reverse primer: 5′-CAGAGGCAGGTGGCTGGT-3′) to obtain an expected product size of 744 bp. The PCR products amplified from untransfected and TALEN-transfected cells were hybridised for heteroduplex formation, followed by digestion with Surveyor nuclease (Surveyor Nuclease Kit, Transgenomics, Glasgow, UK) for the detection of base mismatch at the cut site due to non-homologous end joining (expected product sizes 243 and 501 bp). The digested products were electrophoresed on a 2% agarose gel containing Safeview (NBS Biologicals, Huntingdon, UK; 1:10 000 [v/v] of agarose gel) and detected by exposure to u.v. light.

### Osteoclast formation assay and TRAP staining

Control, GFP-transfected and TALEN-transfected RAW264.7 cells were seeded at a density of 20 000 cells/well in a 48-well plate in a medium containing 50 ng/ml mouse RANKL (#462-TEC, R&D Systems, Abingdon, UK) and cultured for up to 72 hours until multinucleated cells were observed in the control wells. RAW264.7 cells were fixed in 4% [v/v] formaldehyde. Tartrate-resistant acid phosphatase (TRAP) reagent was prepared as described previously (Itzstein & van 't Hof 2012), and the cells were incubated with it at 37 °C for 60 min. The cells were then analysed on an EVOS inverted microscope (Life Technologies). Osteoclast-like cells were defined as those that were TRAP-positive and contained three or more nuclei.

### Generation of Myc- and FLAG-tagged WT and mutant RANK plasmids

**Figure 3:**
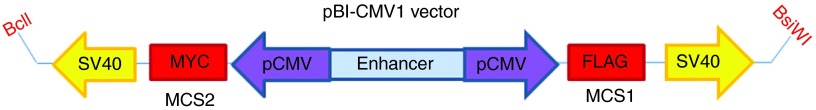
Schematic diagram showing inserted FLAG- and Myc-tagged RANK in MCS1 and MCS2 of the expression cassette from pBI-CMV vector.

Myc- and FLAG-tagged WT and mutant RANK bidirectional expression vectors were generated by a combination of In-Fusion PCR and ligation. WT-RANK, W434X-RANK and G280X-RANK were cloned into the Myc-tagged pcDNA 3.1 (#V855-20, Life Technologies) and FLAG-tagged pCMVTag4a (#211174, Agilent Technologies Inc, Santa Clara, CA, USA). Unique restriction enzyme sites (Bcll and BsiWl) were introduced by site-directed mutagenesis into the pBI-CMV1 vector (#631630, Clontech) and pcDNA5-FRT vectors (#V6010-20, Life Technologies). The pBI-CMV1 vector was digested with Bcll and BsiWl, releasing the bidirectional expression cassette containing the two multiple cloning sites (MCS), two pCMV promoters, two SV40 polyA^+^ subunits and the enhancer (Fig. 3), and this was subcloned into the pcDNA5-FRT plasmid. The FLAG- and Myc-tagged RANKs (WT-, W434X- and G280X-RANK) were introduced into MCS1 and MCS2 respectively by In-Fusion cloning (Takara Bio Europe, Saint-Germain-en-Laye, France) with sequence-specific primers for each of the MCS after linearising the vector with Apal.

### Immunocytochemistry

The expression of exogenous RANK was detected in HeLa cells and HEK293 cells by immunocytochemistry. The cells were seeded on glass coverslips in 48-well plates and incubated overnight. The cells were transfected with the WT-RANKmyc, W434X-RANKmyc and G280X-RANKmyc expression vectors using the jetPRIME transfection reagent (Polyplus transfection, PeqLab, Sarisbury Green, UK) according to the manufacturer's protocol. After 48 h, the cells were fixed in 4% [v/v] formaldehyde. Subcellular localisation of proteins was detected using rabbit anti-Myc antibody (New England Biolabs, Ipswich, UK) and then using Alexa Fluor goat anti-rabbit 555. The cells were counterstained with SYTOX Green (Life Technologies), and the immunostained coverslips were mounted in VECTASHIELD (Vector Labs, Peterborough, UK) mounting medium and observed under a Zeiss LSM 510 confocal microscope (Zeiss, Germany).

### Western blot analysis

HeLa cells were cultured in six-well plates at a density of 1×10^5^ cells/ml, and after 24 h the cells were transfected with WT-RANKmyc, W434X-RANKmyc or G280X-RANKmyc using the JetPRIME transfection reagent. After 48 h, the cells were prepared in a radioimmunoprecipitation assay buffer (RIPA; deoxycholic acid (12.5 mM), SDS (3.5 mM) and IGEPAL (1%) in PBS) containing 1% protease inhibitor cocktail for western blot analysis as described previously (Crockett *et al*. 2011). The proteins on the PVDF membrane were probed with mouse anti-RANK antibody (Imgenex, clone 9A725, #IMG-128A) followed by donkey anti-rabbit-800 secondary antibody (LI-COR Biosciences, Cambridge, UK) and then with mouse anti-Myc antibody (Cell Signaling Technology, Danvers, MA, USA) followed by donkey anti-mouse-680 secondary antibody (LI-COR Biosciences). The blots were analysed on an LI-COR Infrared imager with Odyssey analysis software.

### Immunoprecipitation

HEK293 cells were seeded in 10 cm Petri dishes (4×10^6^ cells/dish) and transfected with Myc- and FLAG-tagged bidirectional RANK constructs using the jetPRIME transfection reagent according to the manufacturer's protocol on the following day. After 48 h, the cells were lysed for immunoprecipitation (IP) after treatment for 24 h, 8 h and 10 min with 40 ng/ml mouse RANKL (R&D Systems).

The transfected cells were lysed on ice in an IP lysis buffer (25 mM HEPES, 150 mM NaCl and 1% [v/v] Triton X-100 in distilled water, pH 7.5, supplemented with 25 mM NaF, 1 mM EDTA, 1 mM sodium orthovanadate, 1% [v/v] protease inhibitor cocktail (Sigma–Aldrich) and 0.25% [v/v] phosphatase inhibitor cocktail (Sigma–Aldrich)). IP was performed on 500 μg of total lysate with mouse anti-Myc antibody (4 μl/sample), and Myc-tagged proteins were isolated using Protein G agarose beads (30 μl/tube).The samples were then analysed by western blot analysis using mouse anti-Myc and rabbit anti-FLAG primary antibodies. The blots were then probed for TRAF6 using a rabbit anti-TRAF6 antibody (Santa Cruz). Blots were analysed on a LI-COR infrared scanner (LI-COR, Cambridge) using Odyssey software.

### Activation of NFκB signalling

HEK293 cells were cultured in a six-well plate and transfected with full-length Myc-tagged WT or W434X or G280X mutant RANK vectors using the jetPRIME transfection reagent. After 2 days, the cells were serum-starved for 3 h and stimulated with mouse RANKL at a concentration of 40 ng/ml for 5 min, 10 min and 1 h at 37 °C. Cells treated with human TNFα (125 ng/ml, PeproTech EC, Ltd, London, UK) for 5 min were used as positive controls. The cells were washed with ice-cold PBS once and lysed in RIPA buffer containing 1% protease inhibitor cocktail. Phosphorylated p65 was detected by western blot analysis using rabbit anti-phospho-p65 (Serine 536) MAB ([93H1] #3033, Cell Signalling Technology) and anti-rabbit infrared-labelled antibody (LI-COR Biosciences) using the Infrared imaging system (LI-COR Biosciences) with Odyssey software. The relative abundance of pp65 compared with p65 in each blot was calculated using ImageJ (Schneider *et al*. 2012).

### Statistical analyses

Data are expressed as means±s.d. from at least three independent experiments. Statistical analyses were carried out using two-tailed Student's *t*-test, and *P*<0.05 was considered statistically significant.

## Results

### TALEN-induced double-strand breaks in the W434X mutation region in *Tnfrsf11a* prevent osteoclast-like cell formation

GFP-transfected RAW264.7 cells were found to be successfully transfected using the Neon electroporation system (Fig. 2Bi), and these transfected cells were found to be capable of forming multinucleated osteoclast-like cells in the presence of RANKL (Fig. 2Bii).

Surveyor nuclease analysis of genomic DNA extracted from TALEN-transfected and untransfected RAW264.7 cells revealed that the double-strand breaks had been successfully introduced into the region surrounding the W434X (W430X in mouse) mutation site. The 744 bp PCR product was cleaved to 501 and 243 bp fragments (Fig. 2C) by the surveyor nuclease, which cuts mismatched heteroduplex DNA (Qiu *et al*. 2004). The band densities were quantified using ImageJ, and the percentage of cleavage was calculated as 53% (density of cleaved products/total product density×100).

### Osteoclast formation assay of RAW264.7 cells

Following incubation for 4 days in RANKL-containing medium, analysis of TRAP-positive multinucleated cell formation revealed a significant (*P*<0.01) reduction in osteoclast-like cell formation in cultures that had been transfected with the TALENs targeted at the region surrounding the location of the W430X (mouse equivalent) mutation (Fig. 2A, D and E), indicating that the introduction of double-strand DNA breaks within this region indeed abolishes RANK function.

### Both W434X-RANK and G280X-RANK are expressed at the plasma membrane

**Figure 4:**
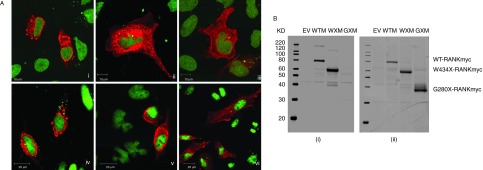
(A) Immunolocalisation of i) WT-RANKmyc, ii) W434X-RANKmyc and iii) G280X-RANKmyc proteins in HeLa cells and of iv) WT-RANKmyc, v) W434X-RANKmyc and vi) G280X-RANKmyc proteins in HEK293 cells. Myc-tagged RANK proteins (red) were detected using rabbit anti-Myc antibody and Alexa Fluor 555 goat anti-rabbit secondary antibody. The nuclei of the cells were counterstained with SYTOX Green (scale bars=10 μm (HeLa) or 20 μm (HEK293)). (B) HeLa cells were transfected with WT-Myc-tagged, W434X-Myc-tagged and G280X-Myc-tagged DNA constructs. Membranes were probed for RANK using i) mouse anti-RANK and anti-mouse-680 and ii) mouse anti-Myc antibody and anti-mouse-800 antibodies. KD, molecular mass markers; EV, empty vector; WTM, WT-Myc-tagged RANK; WXM, W434X-Myc-tagged RANK; GXM, G280X-Myc-tagged RANK.

Immunostaining followed by confocal analysis was performed in HeLa cells to detect Myc-tagged WT and mutant RANK 48 h after transfection. WT-RANKmyc was detected in the Golgi apparatus (Fig. 4Ai), and W434X-RANK and G280X-RANK were clearly detected at the cell surface as well as intracellularly (Fig. 4Aii and iii). The pattern of expression was different between WT and mutant constructs as, in contrast to WT-RANKmyc, the intracellular distribution of G280X-RANK and W434X-RANK occurred throughout the cytosol and was not restricted to vesicular structures. This finding indicated that the truncated RANK proteins were capable of translocating to the cell surface and therefore capable of interacting with RANKL.

### Overexpressed WT-RANK, W434X-RANK and G280X-RANK protein detection by western blot analysis

WT-RANKmyc and W434X-RANKmyc were detected using mouse anti-RANK antibody (the epitope for which is in the cytoplasmic domain between residues 330 and 427 of human RANK) and anti-Myc antibody (Fig. 4Bi), whereas G280X-RANKmyc could be detected only with the anti-Myc antibody (Fig. 4Bii). While WT-RANKmyc migrated at an apparent molecular mass of around 80 kDa, W434X-RANKmyc was detected at 55 kDa and G280X-RANKmyc was detected at approximately 35 kDa.

### Ligand-independent oligomerisation is dependent on the region downstream of position 434 in RANK, whereas ligand-dependent oligomerisation is not

**Figure 5:**
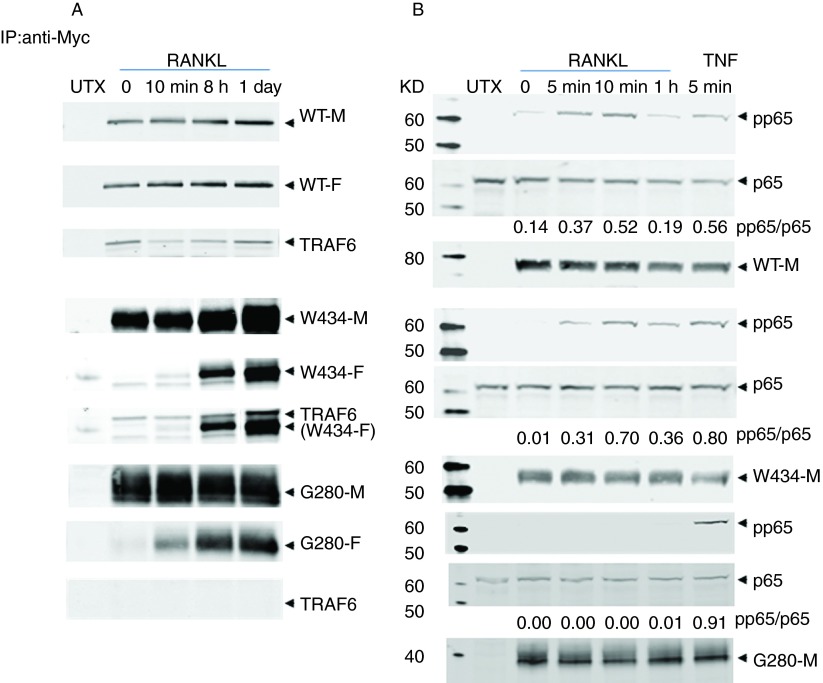
(A) Detection of oligomeric WT-, W434X- and G280X-RANK proteins after RANKL stimulation. FLAG-tagged RANK proteins were detected with an anti-FLAG antibody (and anti-rabbit-680-labelled secondary antibody) following immunoprecipitation using an anti-Myc antibody in cell lysates that had been transfected with WT-RANKmyc (WT-M)–WT-RANK-FLAG (WT-F), W434X-RANKmyc (W434-M)–W434X-RANK-FLAG (W434-F) or G280X-RANKmyc (G280-M)–G280X-RANK-FLAG (G280-F) (W434X-RANK blots were derived from a single blot with additional time points: only bands from 0 min, 10 min, 8 h and 1 day are shown). The blots were then probed with mouse anti-Myc antibody (and anti-mouse-800-labelled secondary antibody) and rabbit anti-TRAF6 (and anti-rabbit-680-labelled secondary antibody). The PVDF membrane was scanned on a LI-COR infrared scanner with Odyssey software. (B) Effect of truncating mutations of C-terminal RANK on the activation of the NFκB pathway. HEK293 cells were transfected with WT- (WT-M), W434X- (W434-M) or G280X-RANK (G280-M) and incubated for 48 h. After 3 h of serum starvation, the cells were stimulated with murine RANKL or 125 ng/ml TNFα. The western blot membrane was probed with mouse anti-phospho-p65, rabbit anti-p65 and mouse anti-Myc antibodies and species-specific infrared-tagged antibodies (680 and 800 nm). The PVDF membrane was scanned on a LI-COR infrared scanner with Odyssey software. Arrowheads highlight relevant bands. Data are representative of three independent experiments. pp65/p65 represents the densitometric ratio of pp65:p65 abundance for each blot shown.

Western blot analysis (Fig. 5A), using anti-FLAG primary antibody, of immunoprecipitated Myc-tagged proteins from cells transfected with WT-RANKmyc–WT-RANK-FLAG vectors revealed that, as expected, overexpressed WT-RANKmyc and WT-RANK-FLAG associate with each other in the absence of RANKL stimulation. When the transfected cells were exposed to RANKL for 10 min, 8 h and 24 h, there was no detectable increase in the abundance of oligomer formation. TRAF6 could be detected in the presence and absence of RANKL, indicating that TRAF6 associates with trimeric RANK in a ligand-independent manner. In contrast, cells overexpressing W434X-RANKmyc–W434X-RANK-FLAG exhibited no oligomer formation in the absence of RANKL, but in the presence of RANKL, the association between Myc- and FLAG-tagged W434X-RANK increased over time. TRAF6 was associated with W434X-RANK in both unstimulated and RANKL-stimulated cells, increasing in parallel with increased oligomer formation. Finally, ligand-independent oligomer formation was not detected between G280X-RANKmyc and G280X-RANK-FLAG, but oligomer formation was stimulated by RANKL treatment. As expected, TRAF6 did not associate with G280X-RANK in unstimulated or RANKL-stimulated cells. Taken together, these results demonstrate that the absence of the PLAD does not interfere with ligand-induced RANK oligomer formation and that TRAF6 binding still occurs in the absence of the most distal TRAF6-binding motif.

### W434X-RANK, but not G280X-RANK, supports RANKL-dependent activation of NFκB

After establishing that ligand-dependent oligomerisation was unaffected in W434X-RANK- and G280X-RANK-transfected cells, the ability of these RANK proteins to support the activation of NFκB was explored (Fig. 5B). In all cells, TNFα induced robust activation of p65 after 5 min. In cells expressing WT-RANKmyc or W434X-RANKmyc, ligand-dependent phospho-p65 activation was detected at both 5 and 10 min after RANKL stimulation with the signal intensity being reduced after 1 h. In cells expressing G280X-RANK, phospho-p65 activation was not detected after RANKL stimulation. In cells expressing WT-RANKmyc, some ligand-independent activation was also detected.

## Discussion

All TNFR family members have a series of cysteine-rich domains (CRD) in the extracellular region, which are the loci for ligand binding (Wiens & Glenney 2011). The extracellular part of the majority of these receptors also contains the PLAD, which is located within CRD1, distinct from the ligand-binding sites present in CRD2/3 (Chan *et al*. 2000). Previous experiments have shown that in the absence of ligand binding, the receptors can oligomerise and are capable of signal transduction due to self-association through the PLAD, whereas in the absence of the PLAD, the ligand is unable to activate receptor signalling, indicating an essential role for the PLAD in TNFR signalling (Chan *et al*. 2000, Siegel *et al*. 2000). Although RANK is a member of the TNFR superfamily that functions as a homotrimer, it has a short sequence (‘IVVY^535–538^’) motif within the intracellular domain that has previously been suggested to function in a way analogous to the PLAD in other TNFRs with a suggested essential role in RANK function (Kanazawa & Kudo 2005). TRAF6 is essential for TNFR-induced NFκB signalling (Ye *et al*. 2002), and RANK contains three conserved TRAF6-binding motifs to activate NFκB (Darnay *et al*. 1998, Galibert *et al*. 1998). Kanazawa and Kudo also highlighted the crucial role of TRAF6 in osteoclastogenesis using a TRAF6 decoy peptide (T6DP) as an inhibitor of TRAF6 activity. The ‘IVVY^535^^–^^538^’ domain was shown to be permissive for murine osteoclastogenesis (Xu *et al*. 2006), and mutations within or the use of blocking peptides against this domain blocked RANKL-induced osteoclastogenesis *in vitro* (Kim *et al*. 2009). Critical for osteoclastogenesis is the establishment of Ca^2^^+^ oscillations necessary for the activation of nuclear factor of activated T-cells 1 (NFATc1) (Takayanagi *et al*. 2002). The region of the RANK cytoplasmic tail around the ‘IVVY’ domain facilitates this activation via interaction with co-stimulatory immunoreceptor tyrosine-based activation motif (ITAM)-containing molecules and the scaffold protein GAB2 (Taguchi *et al*. 2009).

The presence of a PLAD in the intracellular part of the receptor indicates that RANK is, therefore, an exception among the TNFR family members. In this study, the effects of two truncating mutations within the cytoplasmic domain of RANK (W434X and G280X) were investigated in terms of their effect on ligand-independent as well as ligand-dependent oligomerisation and signalling activation. Both mutations result in the deletion of the oligomerisation motif, indicating that if this region is functionally equivalent to a PLAD, then the absence of this domain may be responsible for non-functioning RANK, leading to the severe osteopetrotic phenotype in these patients.

The G280X mutation has previously been shown to inhibit osteoclast formation when cells of a patient were cultured *in vitro* (Guerrini *et al*. 2008). However, the *in vitro* osteoclast differentiation phenotype of cells isolated from a patient carrying the W434X mutation was not investigated. Therefore, we sought to recapitulate this mutation using TALEN to introduce site-specific double-strand DNA breaks into the genome of RAW264.7 macrophage-like cells. RAW264.7 macrophage-like cells are murine cells that express the RANK receptor and from which we can reproducibly generate multinucleated, TRAP-positive ‘osteoclast-like’ cells in the presence of RANKL. We recognise that these cells are not a perfect model for osteoclasts and that clonal variation exists in terms of their ability to form osteoclast-like cells, resorb mineralised substrate and be transfectable (Cuetara *et al*. 2006). For the purposes of the present study, they served as a straightforward model to address the effects of the mutation on the formation of osteoclast-like cells. The TALEN proteins introduced a double-strand break within a 12 bp spacer region centred at position c.G1352, the base position in mouse mRNA equivalent to the location of the c.G1301A (encoding W434X) mutation in human mRNA. The DNA cleavage was sufficient to significantly reduce osteoclast formation in these cultures compared with those that had not incorporated the TALENs, indicating that the introduction of a mutation into this region of the protein ablates osteoclast formation. These results indicate that, in agreement with the low number of osteoclasts in the patient bone biopsy (Guerrini *et al*. 2008), introduction of the W434X mutation prevented *in vitro* osteoclast-like cell formation.

Immunocytochemistry of permeabilised cells revealed that intracellularly, overexpressed WT-RANKmyc was predominantly localised to the Golgi apparatus and its expression was restricted to distinct vesicular structures within the cell. This pattern of expression is similar to that observed in previous studies demonstrating co-localisation of WT-RANK with the Golgi and plasma membrane marker wheat germ agglutinin (Crockett *et al*. 2011) and is consistent with the association between oligomerisation of membrane proteins and retention within the Golgi apparatus (Weisz *et al*. 1993). In contrast, the disruption of the oligomerisation potential within W434X-RANKmyc and G280X-RANKmyc proteins resulted in more diffuse distribution throughout the cells; they did not remain in the Golgi apparatus and were readily detected at the plasma membrane.

Given that the mutant proteins appear to be expressed and localise to the plasma membrane, this left the question as to why patients carrying these mutations exhibit the osteopetrotic osteoclast-poor phenotype *in vivo* and *in vitro*. As the ligand-binding and PLAD regions in RANK are in the extracellular and intracellular regions respectively, it is possible to assess whether the presence of the PLAD was essential for ligand-dependent oligomerisation of the RANK receptor. Our data clearly demonstrate that although the PLAD is required for oligomerisation of the receptor in the absence of ligand, it is not required for oligomerisation in the presence of ligand. This is a different scenario to that observed with other TNF receptor family members where the PLAD is required for ligand-dependent downstream effects (Chan *et al*. 2000) and strongly indicates that pre-ligand assembly is not absolutely required for ligand-induced oligomerisation during RANK-mediated signalling.

RANK cytoplasmic motifs are crucial for the differentiation and survival of osteoclasts (Darnay *et al*. 1998, Galibert *et al*. 1998, Liu *et al*. 2005). In agreement with results from previous overexpression studies (Darnay *et al*. 1998, Crockett *et al*. 2011), we could detect some constitutive activation of NFκB in unstimulated WT-RANKmyc-expressing cells. This ligand-independent activation was not detected in cells expressing either of the truncated proteins.

The G280X mutation in RANK totally abolished the ligand-dependent activation of p65, in agreement with the absolute requirement for TR6AF6 recruitment to mediate RANK signalling (Lamothe *et al*. 2007). This, and not the lack of the PLAD, therefore, explains the lack of osteoclast formation in cells derived from the patient studied (Guerrini *et al*. 2008).

The low-affinity interaction between TRAF proteins and receptor monomers ensures that the membrane-bound inactive receptor monomers do not transduce a signal via TRAFs (Ye *et al*. 2002, Wu & Arron 2003). This is of interest when studying the results of the W434X-RANK experiments. In these immunoprecipitates, TRAF6 was associated with RANK in unstimulated (no oligomerisation) as well as in RANKL-stimulated cells. However, although pp65 was detected in response to RANKL treatment, the absence of pp65 in unstimulated cells demonstrates that it is the process of ligand-dependent RANK oligomerisation that activates TRAF6 to undergo autoubiquitination and subsequent activation of downstream NFκB signalling.

However, this does not explain how the W434X mutation results in an osteopetrotic phenotype. TRAF6 is absolutely required for the proper functioning of osteoclasts (Lomaga *et al*. 1999, Naito *et al*. 1999). In RANK carrying the W434X mutation, the most distal of the three putative TRAF6-binding motifs described in previous studies (Gohda *et al*. 2005, Darnay *et al*. 2007) is absent. Motifs 1 and 2 (Fig. 1) are present, which are capable of supporting NFκB activation and osteoclast differentiation *in vitro*, but only if they are ablated in the context of full-length RANK cytoplasmic tail. Indeed, previous studies examining the effect of TRAF6-binding site ablation in RANK on osteoclast formation examined this by site-specific deletion or substitution of specific motifs/short regions and not in situations of deletion of the distal end of the receptor (Gohda *et al*. 2005, Darnay *et al*. 2007). The key to the osteoclast-poor phenotype associated with the W434X mutation is likely to be a highly conserved region (HCR; between residues 489 and 558) that is not required for short-term NFκB activation, but supports the long-term activation of NFκB and ITAM signals that activate the master osteoclastogenic transcription factor, NFATc1 (Taguchi *et al*. 2009). Our data completely support the hypothesis that association between TRAF6 and the scaffold protein GAB2, which interacts with this HCR, is necessary for osteoclastogenesis (Wada *et al*. 2005, Taguchi *et al*. 2009). Thus, although W434X-RANK activates NFκB through the remaining TRAF6-binding sites (as shown by our results), this activation is not sufficient to support the formation of osteoclasts.

**Figure 6:**
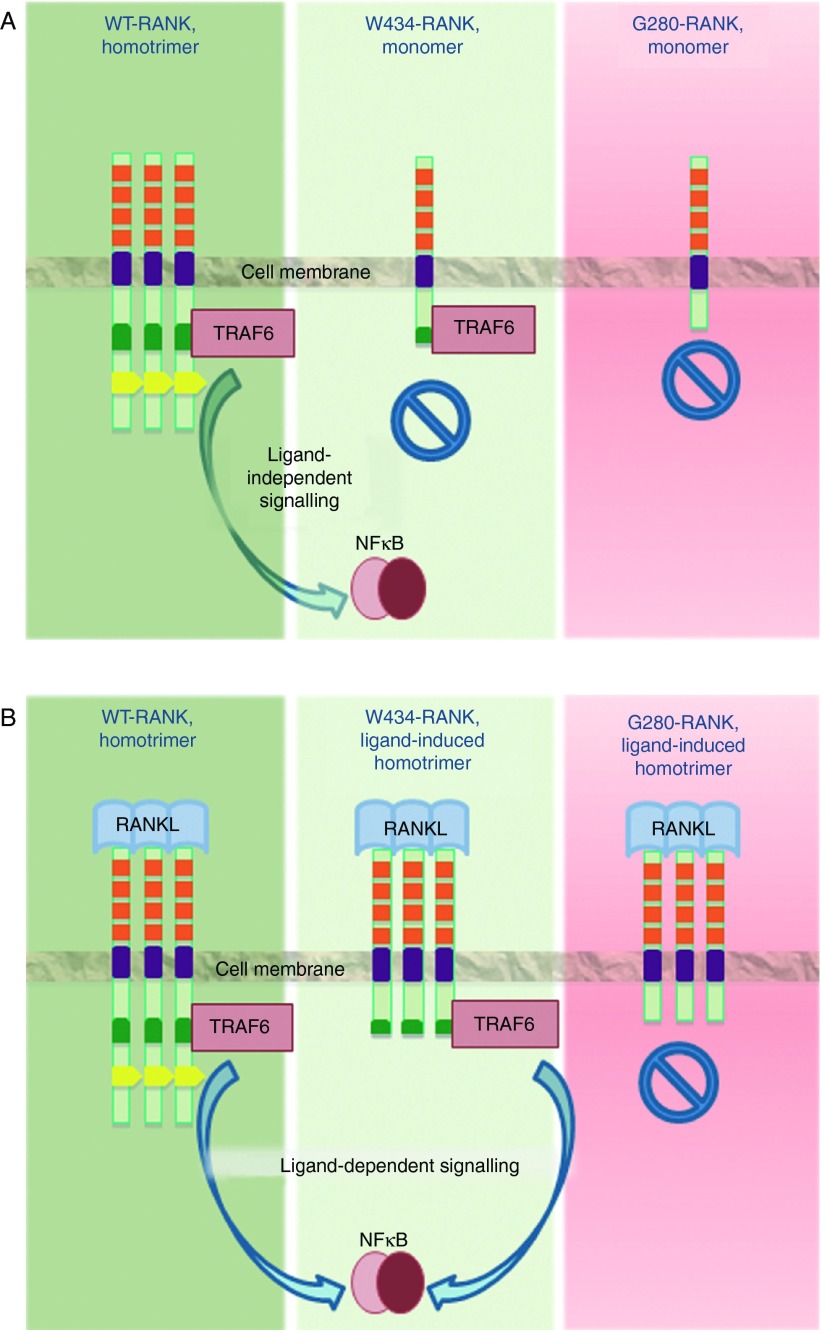
Schematic representation of the findings of the study. (A) Lack of RANK oligomerisation in cells expressing W434X-RANK or G280X-RANK in the absence of ligand. In contrast, cells overexpressing WT-RANK exhibit ligand-independent oligomerisation and it does take place as would be expected of a pre-ligand assembly domain-containing receptor. Both WT-RANK and W434X-RANK, but not G280X-RANK, associate with TRAF6 in the absence of ligand, whereas only WT-RANK displays ligand-independent activation of p65. (B) Oligomerisation of WT-RANK, W434X-RANK and G280X-RANK in response to RANKL. TRAF6 associates with WT-RANK and W434X-RANK, but not with G280X-RANK, and ligand-dependent activation of p65 occurs only downstream of WT-RANK and W434X-RANK.

Taken together, these results provide novel insights into the role of the cytoplasmic oligomerisation motif in the regulation of RANK signalling. The data provide mechanistic molecular evidence for the link between the G280X-RANK and W434X-RANK mutations and the osteoclast-poor osteopetrotic phenotype (Fig. 6). Given the observation that the PLAD is dispensable for signal activation, the data also indicate that targeting the PLAD in RANK as a potential therapeutic for osteoporosis may not be as effective as initially predicted, as highlighted by the limited inhibitory effect of the small peptide (KGDIIVVYVSQT) on the formation of osteoclasts (Taguchi *et al*. 2012).
